# Supplementary material for: Antimicrobial activity of silver-copper coating against aerosols containing surrogate respiratory viruses and bacteria
Source: PLoS One. 2023 Dec 11;18(12):e0294972. doi: 10.1371/journal.pone.0294972 (PMC10712891; doi:10.1371/journal.pone.0294972)
Supplement: S1 Table — (DOCX) [file pone.0294972.s001.docx]

**S1 Table.** Viral and bacterial aerosol supporting information.

| **VIRAL AND BACTERIA AEROSOL ASSAY** | | |
| --- | --- | --- |
| Area of the sample evaluated | 4.90 cm^2^ | |
| Workflow during testing | 0.17±0.009 m/s (testo 405i thermal anemometer) | |
| Aerosol oriented side of sample | Smooth side since the nanocoating was deposited on that side. | |
| Average particle size during the aerosol test | Bacteria particle | *E. coli* (1-3 µm)  *P. aeruginosa* (2-4 µm)  *S. aureus* (0.5 µm-1.5 µm)  *S. epidermidis* (0.5-1.5 µm)  *A. israelii* (1-4 µm)  *A. a. b* (0.5-1.4 µm)  *P. gingivalis* (1-4 µm)  *S. mutans* (1-4 µm) |
|  | Virus surrogates particle | PaMx54 (25 nm)  PaMx60 (25 nm)  PaMx61 (25 nm)  PhiX174 (80-100 nm) |
