# Supplementary figures and images for: Antimicrobial activity of silver-copper coating against aerosols containing surrogate respiratory viruses and bacteria
Source: PLoS One. 2023 Dec 11;18(12):e0294972. doi: 10.1371/journal.pone.0294972 (PMC10712891; doi:10.1371/journal.pone.0294972)

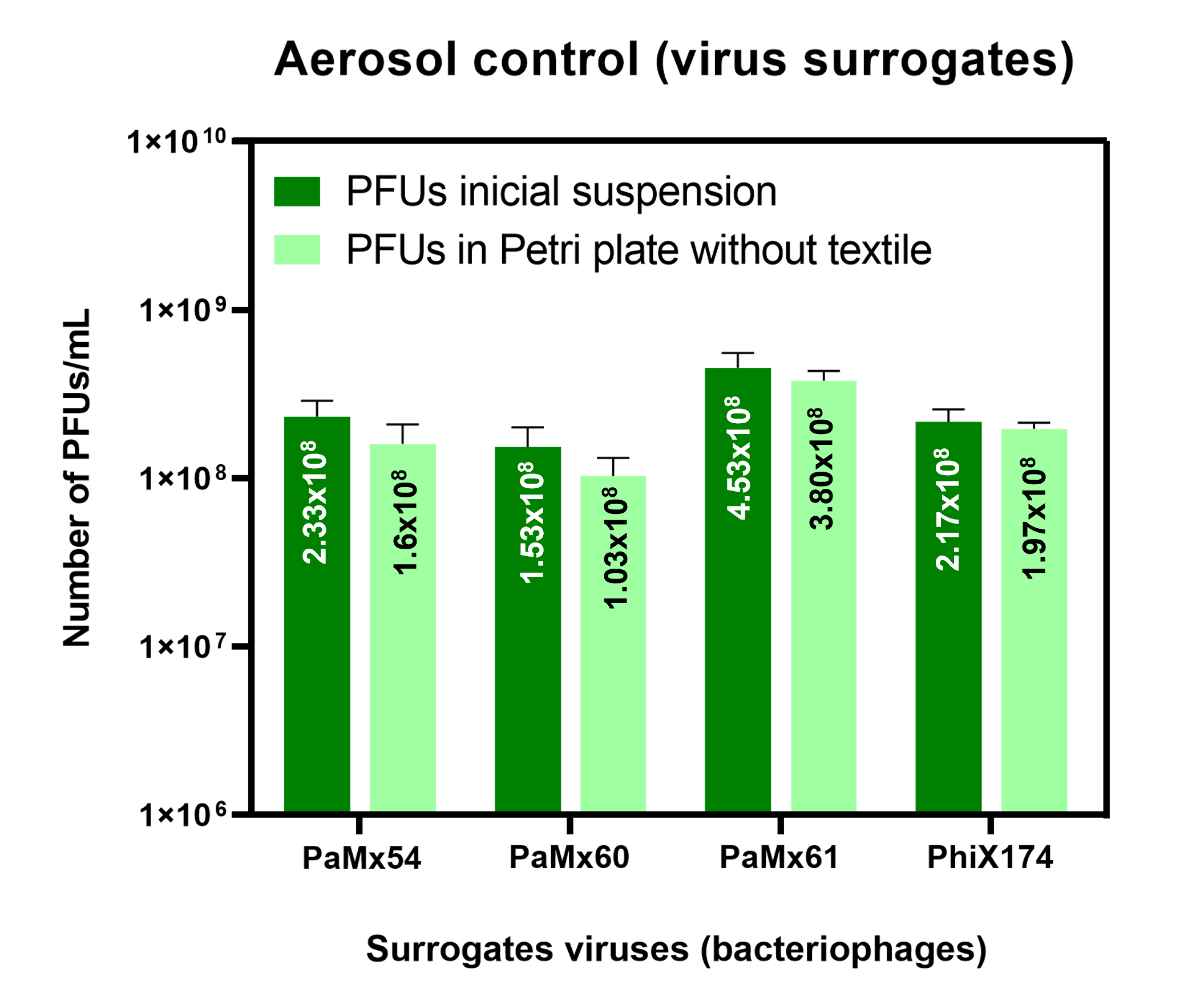

Supplement: S1 Fig — Viral concentration (PFUs) quantified from the agar plates is almost the same as the initial viral suspension. (TIF) [file pone.0294972.s002.tif]

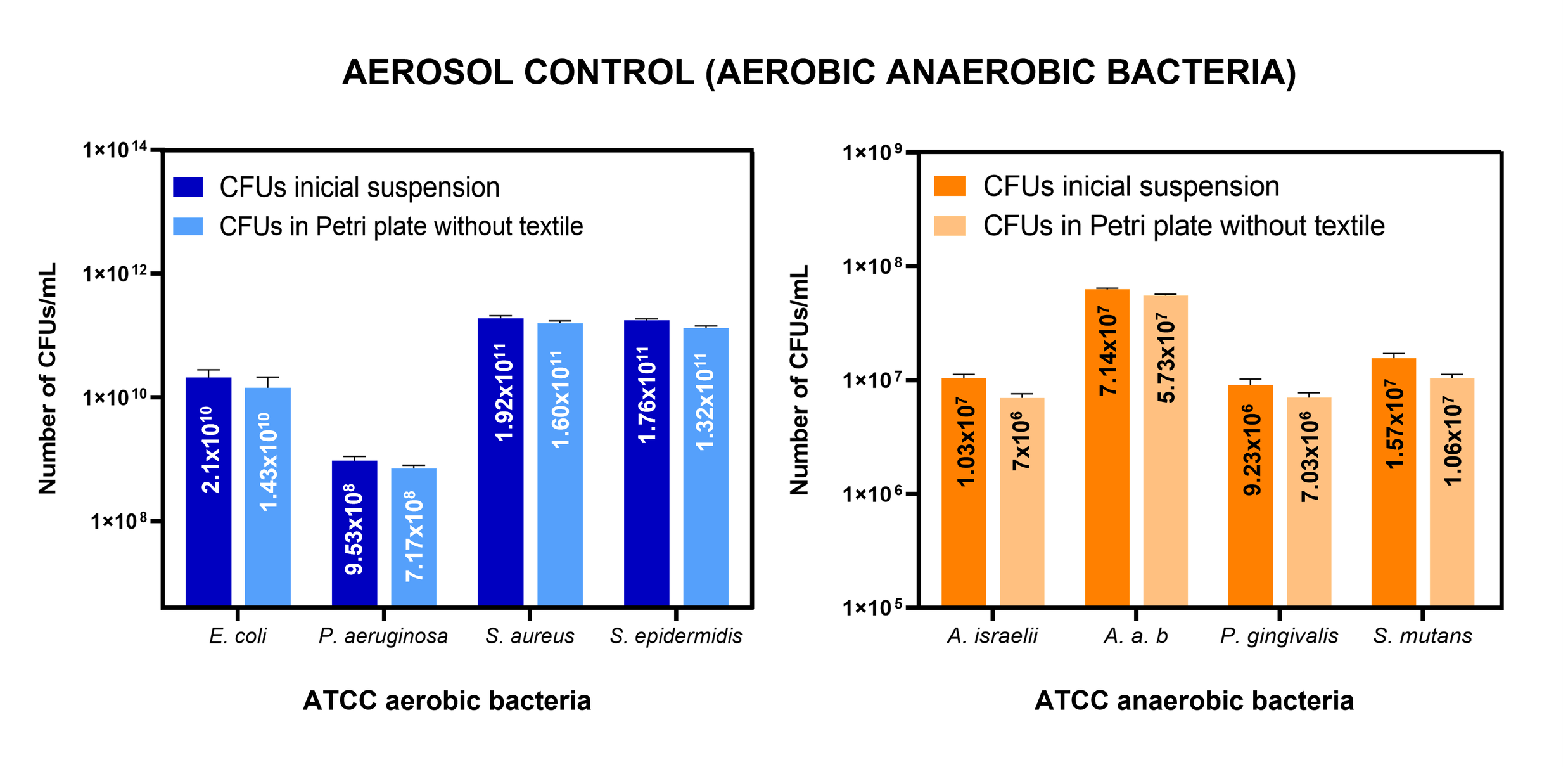

Supplement: S2 Fig — Bacteria concentration (CFUs) quantified from the agar plates is almost the same as the initial bacterial suspension. (TIF) [file pone.0294972.s003.tif]

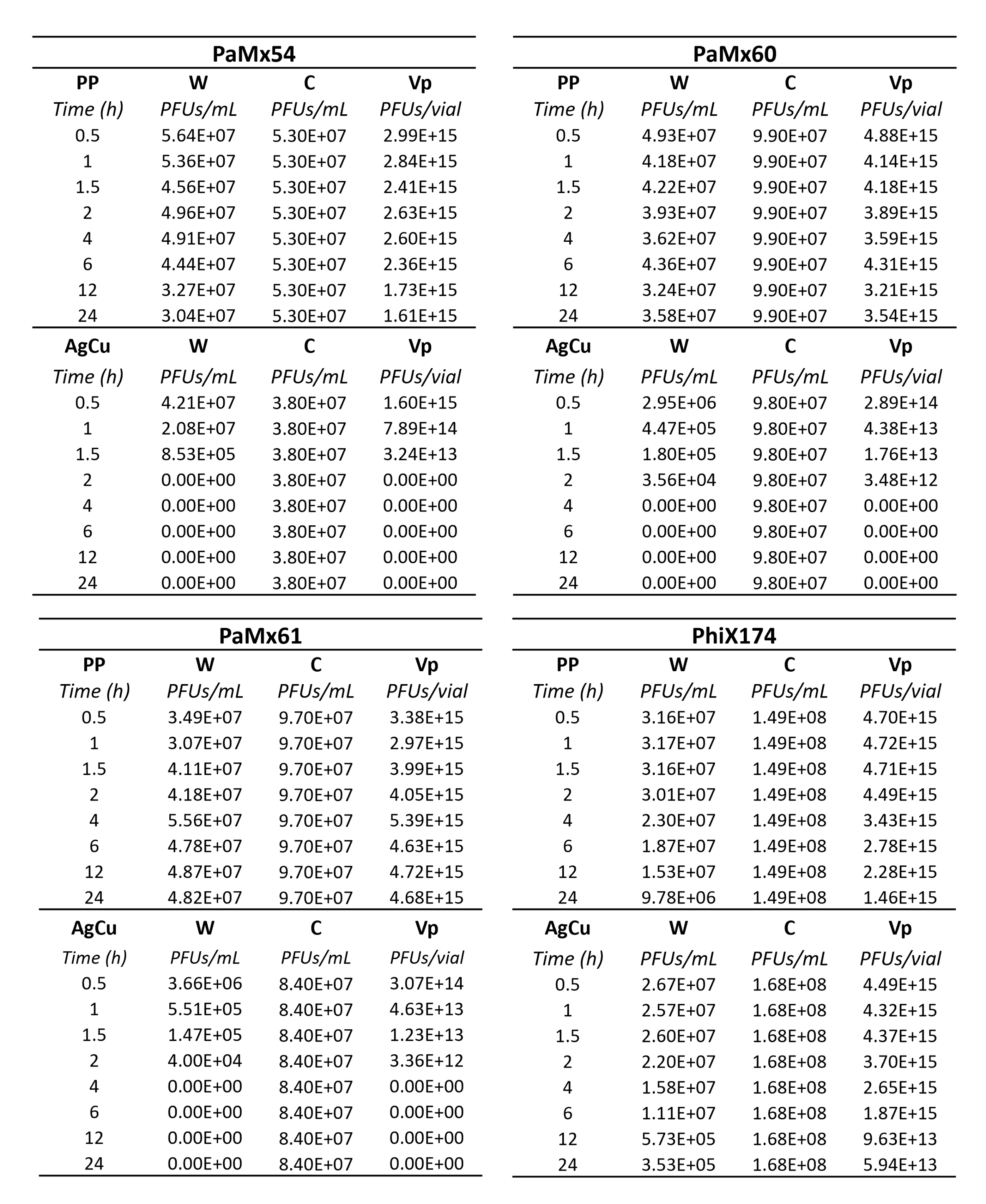

Supplement: S3 Fig — (TIF) [file pone.0294972.s004.tif]

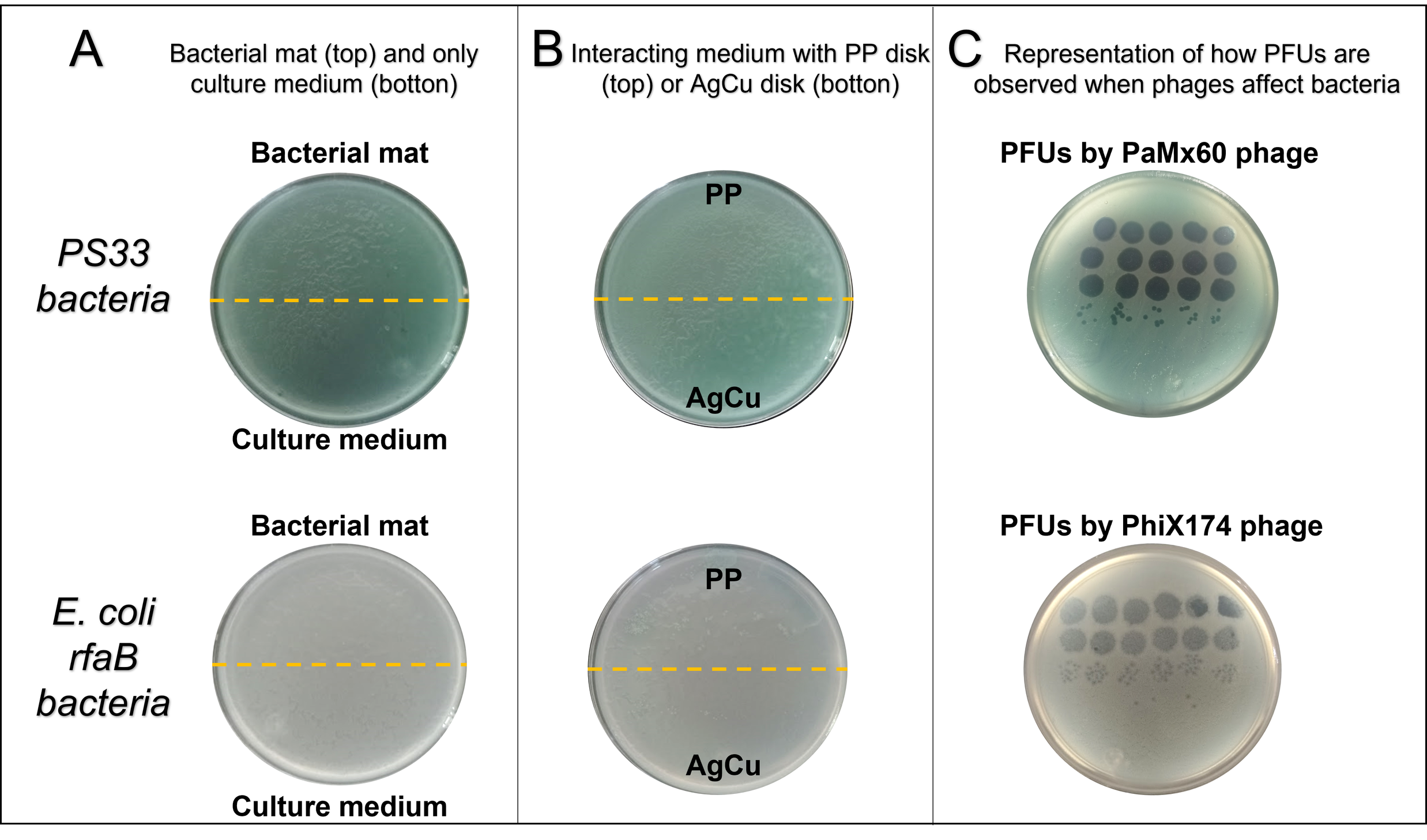

Supplement: S4 Fig — Control experiment to illustrate that the extracts obtained from the uncoated and coated PP do not affect the bacteria host from the surrogate viruses. A. Bacterial mat using only culture media. B. Bacterial mat adding the extracts. C Representative image of the bacteria lysis using the surrogate viruses. (TIF) [file pone.0294972.s005.tif]

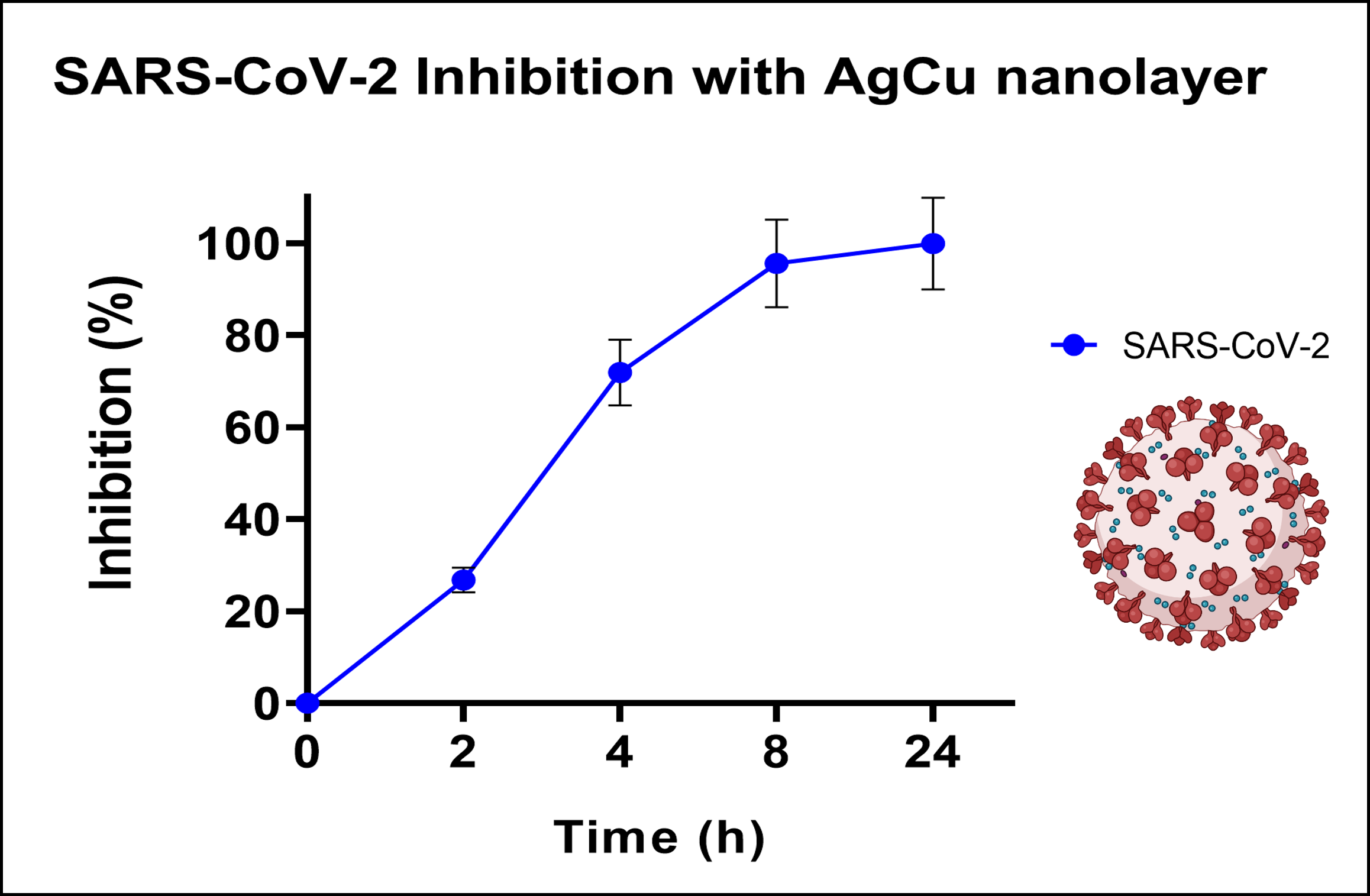

Supplement: S5 Fig — (TIF) [file pone.0294972.s006.tif]

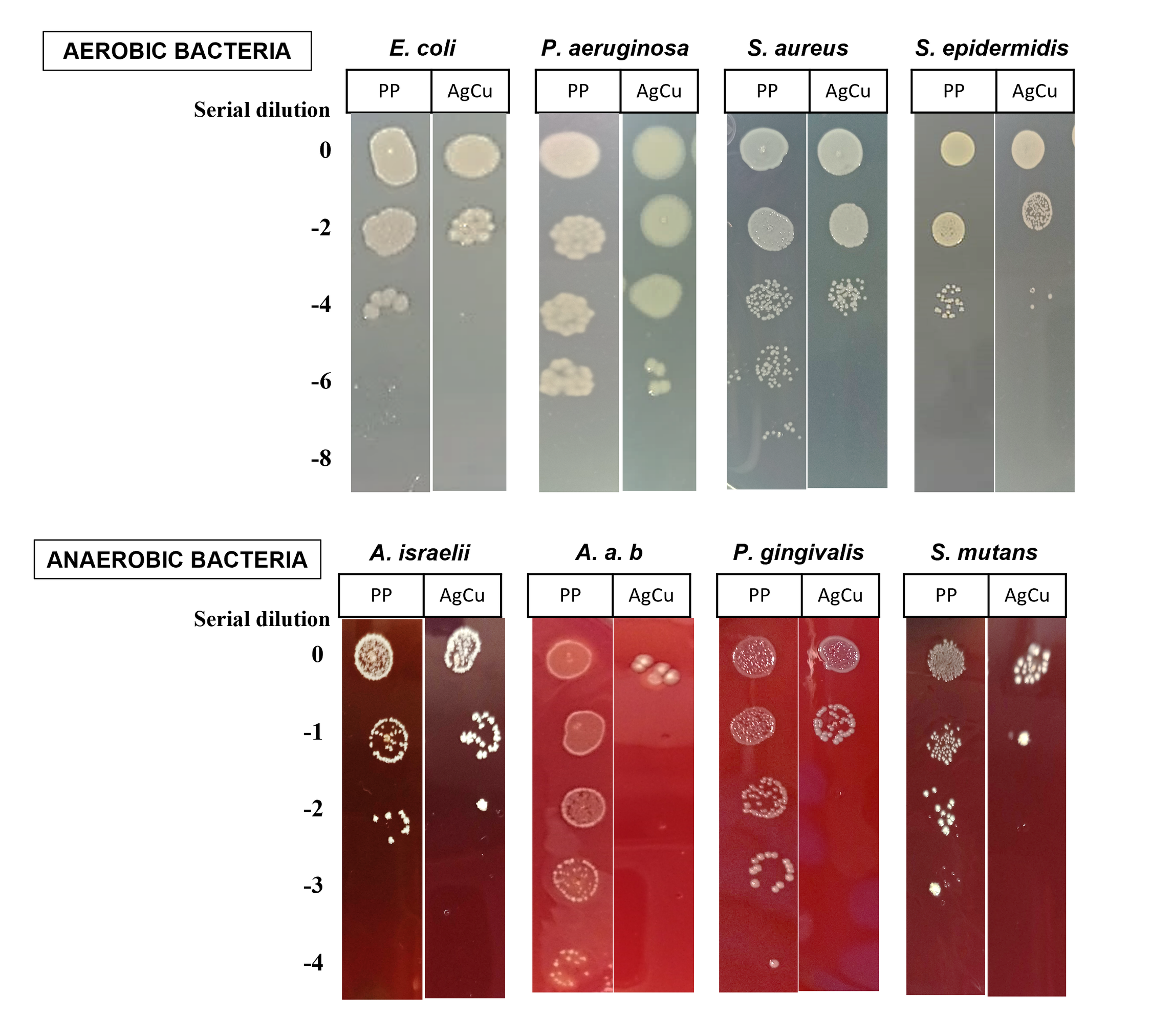

Supplement: S6 Fig — (TIF) [file pone.0294972.s007.tif]
