# Supplementary material for: Antimicrobial activity of silver-copper coating against aerosols containing surrogate respiratory viruses and bacteria
Source: PLoS One. 2023 Dec 11;18(12):e0294972. doi: 10.1371/journal.pone.0294972 (PMC10712891; doi:10.1371/journal.pone.0294972)
Supplement: Attachment — Submitted filename: PONE-D-23-20447 Reviewer Report.pdf [file pone.0294972.s008.pdf]

Reviewer Report

Overview

The work is within the scope of the publication. It explores the use of nano-coating of copper and silver alloy on polypropylene fiber as a material to reduce the activity of various viruses and bacteria of interest. The novelty is the formation of a homogeneous coating and the larger scope of viruses and bacteria studied. The work is comprehensive in demonstrating the inactivation of the microbes used in the study. It is well structured and defines the methodologies well. Some minor revisions on the fabrication of the coating, discussion on the results and the conclusion would further improve the quality of the work. Hence the recommendation is to accept the publication with minor revisions.

Introduction

The introduction is well structured, initially explaining the motivation of the study. Furthermore, it summarizes the current work on antibacterial materials and their major conclusions. Minor Revision: Add appropriate citation in line 72 where different viruses are claimed to be transmitted mainly through aerosols.

Materials and Methods

AgCu nanolayer deposition and characterization

- Minor Revision: The methodology to create the nanocoating is well described. It would additionally be good to add the thickness of the PP used.
- Minor Revision: Moreover, it would be good to understand how deep within the PP does the coating deposit. Hence SEM and EDS at multiple thicknesses till no AgCu is detected within the fiber would be useful but is optional.

All other methods

Overall the methods section is well written with description of key parameters used for the data collection. The figures are helpful in the understanding the methods.

Results

The observations are clearly mentioned and the figures are legible and clearly delineate the key results.

- Minor Revision: In line 315 describe in which locations and in how many locations was the EDS collected.

- Minor Revision: The x-axis for figures 5 and 7 incorrectly spell inhibition as ”inibition”.

Discussion

The discussion explains the key results and elaborates well, on the different mechanisms of antibacterial materials.

- Minor Revision: A potential mechanism for less activity of PhiX174 virus should be elaborated based on understanding the cited work (citation 54 and 56).
- Minor Revision: Line 451-4522 mentions ”The oral anaerobic species exhibited a higher sensitivity to the AgCu nanolayer, which is an interesting finding”. It should be clarified what makes this observation interesting. Example: Is it the first time this has been demonstrated?

Conclusion

Minor Revision: Conclusion should be more quantitative to describe key findings. For example the statement ”It exhibits an exponential reduction in viral load within a short timeframe, highlighting its efficacy in combating airborne respiratory viruses” should mention the timeframe quantitatively (can use any specific virus studied or mention the range).
